# Jointly structuring triadic spaces of meaning and action: book sharing from 3 months on

**DOI:** 10.3389/fpsyg.2014.01390

**Published:** 2014-12-10

**Authors:** Nicole Rossmanith, Alan Costall, Andreas F. Reichelt, Beatriz López, Vasudevi Reddy

**Affiliations:** ^1^Centre for Situated Action and Communication, Department of Psychology, University of PortsmouthPortsmouth, UK; ^2^Cognition and Action Lab, Centre for Neuroscience Studies, Queen's UniversityKingston, ON, Canada

**Keywords:** infant development, intersubjectivity, triadic interaction, action coordination, joint-attention, participatory sense-making, picture book, longitudinal studies

## Abstract

This study explores the emergence of triadic interactions through the example of book sharing. As part of a naturalistic study, 10 infants were visited in their homes from 3–12 months. We report that (1) book sharing as a form of infant-caregiver-object interaction occurred from as early as 3 months. Using qualitative video analysis at a micro-level adapting methodologies from conversation and interaction analysis, we demonstrate that caregivers and infants practiced book sharing in a highly co-ordinated way, with caregivers carving out interaction units and shaping actions into action arcs and infants actively participating and co-ordinating their attention between mother and object from the beginning. We also (2) sketch a developmental trajectory of book sharing over the first year and show that the quality and dynamics of book sharing interactions underwent considerable change as the ecological situation was transformed in parallel with the infants' development of attention and motor skills. Social book sharing interactions reached an early peak at 6 months with the infants becoming more active in the coordination of attention between caregiver and book. From 7 to 9 months, the infants shifted their interest largely to solitary object exploration, in parallel with newly emerging postural and object manipulation skills, disrupting the social coordination and the cultural frame of book sharing. In the period from 9 to 12 months, social book interactions resurfaced, as infants began to effectively integrate manual object actions within the socially shared activity. In conclusion, to fully understand the development and qualities of triadic cultural activities such as book sharing, we need to look especially at the hitherto overlooked early period from 4 to 6 months, and investigate how shared spaces of meaning and action are structured together in and through interaction, creating the substrate for continuing cooperation and cultural learning.

## Introduction

How do we arrive at a shared world? We jointly act in, communicate about, transform and co-create our world. In the process, we smoothly navigate and build complex networks of meaning-making involving persons, objects, and symbols. How do children grow in and into culture? How do they become competent participants in cultural practices, in networks of meaning-making including people and artifacts?

Researchers interested in cultural and social learning mostly start looking from the end of the first year, a period often characterized as a major shift, even revolution (“secondary intersubjectivity” Trevarthen and Hubley, 1978; “9 month revolution” Tomasello, 1999) in development, when infants engage in a number of qualitatively new ways of interacting such as jointly labeling things, following instructions, imitating acts on objects, or frequent gaze checking with their parents. At this point infants are credited with engaging in true triadic interactions, and are considered capable of coordinating for the first time their engagements with objects and their engagement with people. The transition is often seen as the convergence of two lines of development considered to be separate before this point: dyadic infant-caregiver communication and infant-object interaction. This convergence is supposedly mediated by a newly emerging capacity for visual joint attention only then giving rise to conventional labeling and language use, conventional object use and symbolic activities in general, often associated with cultural learning. Interestingly, the seminal studies which constitute much of the empirical basis of this developmental narrative (Trevarthen and Hubley, 1978; Hubley and Trevarthen, 1979; Bakeman and Adamson, 1984), document early modes of combined social and object engagement termed joint praxis and passive joint engagement, respectively. Looking at the data reported, the studies actually show a gradual rather than revolutionary shift toward active triadic engagement on the part of the infant. Hubley and Trevarthen describe how caregivers first introduce their own body (games of the person) and later objects (marking and animating them) as a third pole into their social engagement with their infants. Adamson and Bakeman (1984) document how caregivers change their marking of objects over the course of the first year toward more conventional forms. These data have begun to be picked up on only very recently (De Barbaro et al., 2013; Nomikou et al., 2013; see also Moro and Rodríguez, 2004; Zukow-Goldring, 2012) The standard narrative has also recently been challenged by experimental studies documenting aspects of labeling, and joint attention in infants already at 6 months (Striano and Reid, 2009; Bergelson and Swingley, 2012).

Here we take book sharing as a model activity to explore the development of triadic infant-caregiver-object interactions. In a longitudinal study looking at infants' everyday life activities from 3 to 12 months, this activity turned out to be one of the earliest social interactions involving a complex object, occurring from as early as 3 months.

This early occurrence raises the question: how can infants who are preverbal, do not yet understand the referential character of pictures, and—supposedly—do not have command of joint attention, meaningfully participate in a book sharing activity? As one of the earliest jointly practiced cultural object routines, book sharing provides an excellent model for exploring (1) how a joint object activity is practiced and sustained between asymmetric interaction partners; (2) as an inherently semiotic activity, involving the guiding and mutually orienting of attention, and shared meaning, it allows us to explore how triadic interactions involving mutual coordination and orientation toward common points of reference develop over the first year of life.

While there is an extensive literature on picture book sharing, most studies start looking toward the end of the first year (Ninio and Bruner, 1978; Fletcher and Reese, 2005; but see Van Kleeck et al., 1996), and primarily focus on educational achievements associated with the cultural technology of book reading such as labeling and word learning, picture understanding, and literacy skill.

Here we focus on how the activity of book sharing unfolds, how caregiver, infant, and book respectively guide, sustain, and constrain the unfolding interaction. Taking the interaction as our level of analysis, we draw—in addition to approaches from developmental psychology—on concepts from embodied, situated, dynamical and enactive cognitive science (Fogel, 1993; Thelen and Smith, 1994; De Jaegher and Di Paolo, 2008), adapt methods from ethnography, conversation and interaction analysis (e.g., Goodwin, 2000; Alač, 2005; Streeck et al., 2011; Deppermann, 2013) and use qualitative micro-analysis to explore how, from the interplay of multiple modalities, shared spaces of meaning and action are created around objects and change over time.

## Materials and methods

The book sharing activities documented in this paper have been collected as part of a naturalistic longitudinal study investigating the development of triadic infant-caregiver-object interactions over the first year of life especially focusing on conventional practices and encounters with everyday objects. Ten infants were visited in their homes once a month from 3 to 9 months of age and 7 of them up to the age of 12 months. A smaller pilot study with 6 infants at 3, 4, 5 as well as 9 months of age (3 located in Vienna, 3 in the UK, 4 girls, 4 first ones, 2 of them girls) was conducted in advance of the main study.

### Participants

Of the 10 families participating in the study, 7 were from the UK and 3 from Austria. They were recruited from a wider circle of friends and family acquaintances, from mother and infant groups, as well as through word of mouth and flyers. All infants were living in middle class households with two caregivers and were raised in a monolingual (English or German) environment except one boy raised bilingually in German and Russian. The primary caregivers (mothers in all cases) all had tertiary education and took an active interest in supporting the infant's education. Six of them (all in the UK) returned to either part time or full time work during the course of the study. Of the 10 infants 5 were female and 3 (2 boys and 1 girl) were first born. None of them had medical or cognitive problems.

### Home visit observation procedure and data collection

A typical home visit lasted 3–4 h, spanning 1–2 sleep-wake cycles of the infants. One to two observers accompanied infants and caregivers with a video camera (Panasonic HC-V500 in iframe format: 960 × 540 pixels resolution, 25 frames per second) documenting their everyday activities as they unfolded. For static situations a tripod camera mount was used, though for a large number of cases we switched to a handheld camera approach to capture dynamic scenes especially after infants became mobile. Also, field notes were taken detailing the behavior of the infants, caregivers and siblings, including object and socially directed behavior, layout of the environment, and availability of objects such as toys and tools. In addition, reports from parents were collected giving additional background information on object use. The study was approved by the Psychology Research Ethics Committee of the University of Portsmouth, and was conducted in accordance with the 1964 Declaration of Helsinki and the Code of Human Research Ethics of the BPS. Parents provided written informed consent for the study.

### Data management and analysis

From these raw data, 300+ hours of video recordings, a video library was constructed in Final Cut Pro X (Apple Corporation). Episodes were tagged with keywords organizing activities into basic ecological activity categories, including *(breast) feeding, diaper change, “witnessing,” soothing, social and/or object play, book sharing, sibling interaction, watching TV*. In addition, infant-caregiver-object interactions as well as mutual coordination and orientation episodes were marked. For the purposes of this paper, “book sharing” was selected as a model activity for investigating the development of participation in joint cultural activities and coordination of triadic engagements.

In total 124 book interaction episodes (excluding 15 infant-researcher interactions) were identified and described. For an episode to be counted as a book interaction infants needed to be engaged with a book for at least 30 s. If after a period of disengagement—seen here as an integral part of (especially joint) activities (Stern, 1971; Brazelton et al., 1974; Tronick, 1989; De Jaegher and Di Paolo, 2007)—re-engagement did not occur within 30 s, the book interaction was considered to have ended at the point of disengagement. For all episodes, the actors (infant, mother, father, sibling, …), actions and objects used (types of books), as well as spatial configuration were cataloged.

We distinguished between 2 different types of book interactions: (1) social book sharing (72 episodes), and (2) solitary book exploration (52 episodes). For a book interaction to count as social book sharing the participants each had to be engaged with the book (via gaze or other book oriented actions, e.g., grasping, pointing to, or verbally referencing a page) and to co-ordinate their engagement, that is, to adjust their behavior in response to and in anticipation of each other's—book or partner directed—actions (Bühler, 1927/2000; Fogel, 1993; De Jaegher and Di Paolo, 2007). For each type of book interaction, the number of occurrences and duration of the episodes was determined across ages and families, and basic analysis and visualization was performed using Python (numpy, scipy, and matplotlib packages, free software).

### Qualitative micro-analysis of selected episodes

Of the 72 social book sharing episodes, 20 episodes were selected for further qualitative analysis using the following criteria: (a) only caregiver-infant interactions without siblings to reduce complexity, (b) sampling of interactions from every age group, and (c) richness of interactions including attention and action coordination and communication. These selected episodes were transcribed and analyzed drawing on methods from conversation analysis and interaction analysis, adapted to the study of preverbal infants, with a special focus on embodiment and multimodality (Goodwin, 2000; Alač, 2005; Demuth, 2012; Deppermann, 2013). The analysis was performed in ELAN (free software, The Language Archive, Max-Planck-Institute for Psycholinguistics, Nijmegen Brugman et al., 2004) with audio pitch and intensity extraction performed in Praat (free software, by Paul Boersma and David Weenink, University of Amsterdam).

The videos were repeatedly viewed and described in an iterative process looping back and forth between video and transcript (using ELAN), including gross description, and particular tiers for vocalization, audio pitch and intensity, action and gaze of caregiver and infant. Thus a multi-tiered, parallel record of the episode was constructed and visualized similar to a music score sheet, mapping a range of descriptors to the video stream and relating them to each other in time. Using these visualizations, we analyzed the sequential organization of the actions and how the various strands of an action, spanning multiple modalities, relate to each other and play together in the coordination of action. Transcripts were compared across infants and ages. Some transcription and video stills from ELAN are also used for purposes of illustration.

## Results and discussion

### General results: population level results, the “Umwelt” of the infants and three book sharing examples

#### Population level results

Book sharing was practiced in all 10 participating families (ranging from 2 to 20 episodes per infant). We documented the activity from as early 3 as months (4 families) right from the beginning of the observation period, and no later than by 6 months for all families. To our knowledge, this is the first time book sharing interactions at this early age have been described in the literature.

**Figure 1:**
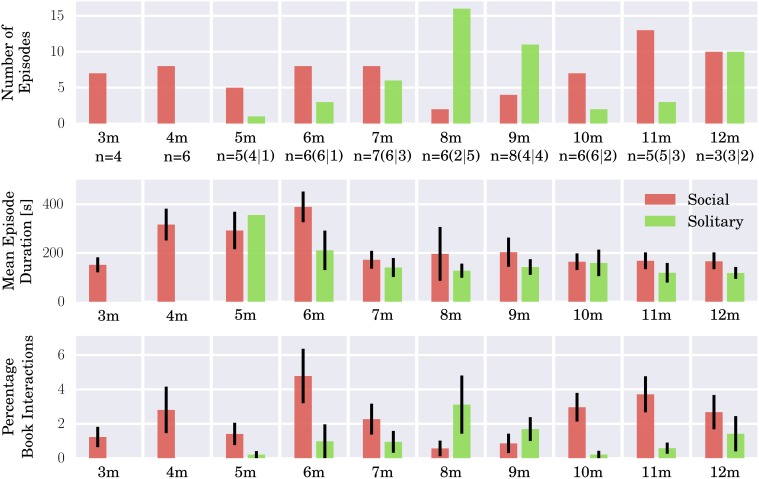
**Social book sharing interactions (red) and solitary book exploration episodes (green) for all infants from 3–12 months**. **Top:** Number of episodes for each month. In addition, the absolute number (“*n* =”) of individual infants represented in the sample (out of the total of 10) is also given below the bar graph, first in total and then in brackets for social and solitary interactions, respectively, as one infant may engage in multiple episodes in the course of a home visit. **Middle:** Mean episode durations for each month with standard errors of the mean (SEM). **Bottom:** Total book interaction time expressed as percentages of the total recorded time infants were awake at each month averaged over all infants. The figure gives an overview of the distributions of the documented book sharing episodes which form the basis of the qualitative study. Note the small sample size.

Social book sharing provided the context for infants' first encounters with books. Later, in the second half of the first year, they also began to approach and interact with books on their own in solitary book exploration. Figure 1 (top) shows the number of occurrences of book interaction episodes for all infants observed in the longitudinal study, by age group and type (social or solitary). Note that we include these data to give an overview of the distribution of episodes forming the basis for the qualitative study. Also note the overall small sample size and that key variables such as the frequency of book sharing offers, and presence and comparability of books in the environment were not controlled for in the naturalistic study as would have been the case in an experimental study. Throughout we focus on two relatively robust measures to complement insights about the changing nature of book interactions gained from qualitative analysis: (1) the relative prevalence of social vs. solitary book interactions, and (2) the changes in mean episode duration over the course of the first year. While social book sharing interaction occurred from as early as 3 months, solitary book exploration episodes started to occur at around 6 months, displacing social book sharing as the dominant type of interaction at 8–9 months. From around 10 months on, social book sharing interactions became dominant again until a balance was reached at 12 months. Figure 1 (middle) shows the mean durations (in seconds) of book sharing episodes for all infants, by age group and type. Starting from durations of around 2 and a half minutes at 3 months, mean durations increased considerably from 4 months reaching a peak of over 6 min at 6 months. From 7 months on, mean durations showed a sharp decrease, as book sharing interactions dropped by more than half to around 3 min duration and then stayed relatively constant. Social and solitary book interactions accounted for from around 1% (at 3 months) to around 5% (at 6 months) of the total recorded time that infants were awake on average at each month as shown in Figure 1 (bottom), with their distributions largely reflecting the overall trend from social to solitary to balanced book interaction and the reduction in mean episode duration after 6 months.

#### The “Umwelt”^1^ of infants at 3–4 months of age

Before turning to the book sharing interactions in detail, we provide a sketch of the larger context of everyday life with a 3–4 month old infant as it presented itself in the study and is described in the literature. How do infants engage with their world at 3–4 months and what does their world look like at this age? At 3 months of age, infants are getting more and more interested in their surroundings. They have good control over their gaze (with a well developed oculomotor system) and increasingly look at and track objects in their environment (Von Hofsten and Rosander, 1997) Apart from that, however, their possibilities for effectively interacting with their world are quite restricted: they are able to hold and move their head, but are not yet able to support their body, turn or move about. Accordingly, the infants in the study at this age spent a lot of time either in a supine position, lying on their backs, or in a reclined sitting position with their backs supported in a baby rocker. In accordance with their postural capacities, they were able to perform coordinated whole body movements, reach toward and start hitting objects, but were not yet able to effectively grasp, mouth or manipulate objects (for a review of the developmental trajectories of motor skills see Adolph and Berger, 2011).

At 3–4 months infants are, however, already fluent conversation partners: by then, they have already actively participated in dyadic proto-conversations with their caregivers for several weeks, fully utilizing and practicing all their capacities including gaze and facial expressions, vocalizations, and rhythmic coordinated whole body movements (Trevarthen, 1974; Bateson, 1975, 1979; Snow, 1977; Bullowa, 1979; Masataka, 2003). Not only are they aware of the dialogical, mutual give-and take character of the interaction—getting upset when the mother's face became unresponsive (Tronick et al., 1978) or when confronted with a friendly but non-contingent (playback) response (Murray and Trevarthen, 1985)—but they are able to regulate their own state of arousal as well as the course of the interaction by turning their gaze and head toward or away from the caregiver (Stern, 1971) and even seem to be able to place their own vocalization exactly at the right time and place at the right pitch in jointly created vocal phrases (Malloch, 2000; Malloch and Trevarthen, 2009).

As infants now take a wider interest in their surroundings (Trevarthen and Hubley, 1978),—in tandem with their increased waking and attentional periods—while still lacking the means to pursue their active interests, to explore or manipulate the world on their own—they pose a new set of challenges and opportunities to caregivers. Therefore, at this stage a large part of caregiving activities observed in the longitudinal study—apart from feeding, diaper change and putting them to bed—was to keep infants content and “entertained”: the caregivers in the study responded to this challenge both by taking the infant to the world and by bringing the world to the infant. They did the former by taking the infants along with them, when doing their daily chores, e.g., placing them in a baby rocker, so they had a good view of the activities, regularly addressing them and bringing household objects or food items to their attention (e.g., rhythmically moving and labeling them) and occasionally also within their reach. They did the latter through presenting, looming and animating everyday life objects as well as specifically designed toys. Caregivers also placed them in specifically designed environments such as activity mats and baby-gyms where they were able to interact with objects dangling from toy bars. In contrast to their previous exposure to only a small range of objects, a whole range of new and manipulable objects now enter the infant's world.

Thus infants were introduced to objects very early at 3–4 months in the context of social interactions. This was also the context in which infants first encountered picture books and book sharing, which took 2 different forms: (1) Their caregivers directly engaged them with books, often specifically designed for young infants. (2) They took part in the picture book reading activities of older siblings and caregivers.

#### Three examples of early book sharing interactions

**Figure 2:**
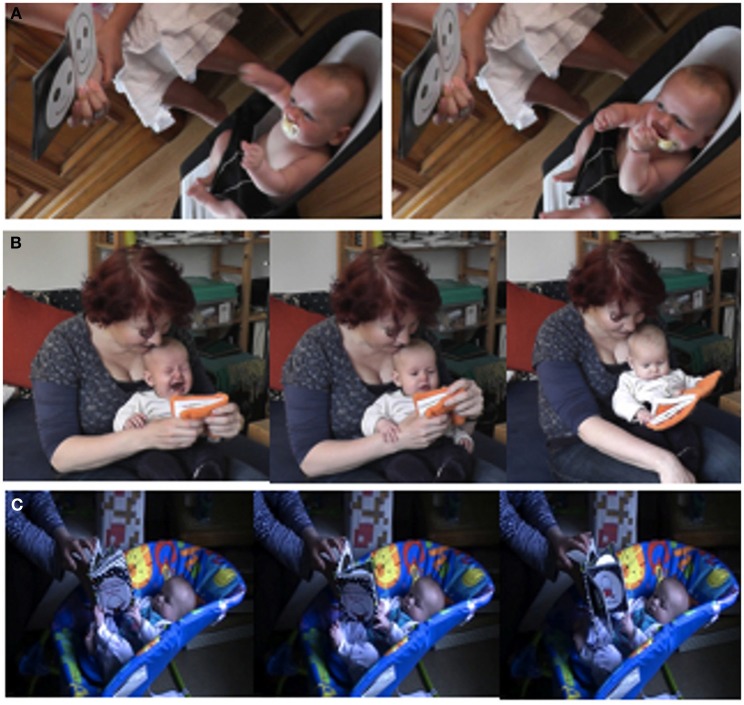
**Three examples of book sharing with books specifically designed for young infants. (A)** Visually engaging a 3-month-old with high contrast patterns. **(B)** Soothing a 3-month-old with crinkly pages. **(C)** Scaffolding a 3.5-month-old's motor skills with rigid pages.

Figure 2 shows three instances of very early book sharing with 3-month-olds. Example A shows a 3-month-old boy vocalizing toward a black and white high contrast face pattern in a book specifically designed to engage very young infants, even new-borns, to meet their particular skills, needs, and interests. In the second example, B, a mother is rustling the crinkly pages of a brightly colored book to soothe her crying 3-month-old daughter. As the infant abruptly stops crying, she begins to engage her daughter in more conventional book sharing, drawing attention to pictures, turning pages, and inviting participation. The infant now and again grasps, holds onto, and crumples the soft pages producing more crinkling noise. In example C, after demonstrating page turning as an action of suspense and release—when a new page is revealed—the book is presented and held in place within the reach of the infant. The book with its rigid pages, solidly bound together at one end, provides a stable structure to interact with that is still highly flexible with easily movable parts along a single degree of freedom. This allows the infant not yet able to properly grasp an object to nevertheless effectively turn pages, thus exerting control over his sensory stimulation.

These three book sharing episodes are examples of early infant-caregiver-object interactions in everyday life, where the object—the book—plays a central role in the interaction. These books have been specifically designed to meet the infants' needs: their physical properties are adapted to the infants' perceptual capacities (high contrast patterns, crinkly pages), and serve as a scaffold for their rudimentary motor skills (rigid pages). In contrast to conventional books, this design emphasizes the effective interaction with the medium, the physical properties of the book and pragmatic actions performed on them. The specifically designed books serve as a bridge between the capacities and needs of infant and caregiver, as well as between caregiving and the cultural practice of reading. Indeed, in all three examples specific material aspects present in the book also capture and afford some of the general, mainly pragmatic aspects of conventional book reading: the format of the book itself is present, as is the format of the activity that has a definite beginning and end corresponding to working through a book from cover to cover, as well as the activity of page turning. Even more, already at 3–4 months, infants regularly experienced episodes involving the full range of book sharing typical for older children including more conventional, complex, and semiotic aspects such as pointing, content labeling, as well as reading and narration (Fletcher and Reese, 2005) as will be discussed in more detail in the next section.

### Early occurrence of smoothly coordinated book sharing interactions at 3–4 months of age

Given young infants' inability to interact with objects on their own yet—in contrast to their active role in proto-conversations—and the widely held theoretical view that they are not yet able to co-ordinate their engagement between people and objects (Hubley and Trevarthen, 1979; Bakeman and Adamson, 1984; Carpenter et al., 1998; Tomasello et al., 2005) the question now arises: How do book sharing interactions work at a micro-level, how do they unfold over time? How are they initiated and sustained, and what are the respective roles of the participants?

#### The contribution of the caregivers: establishing contact, carving out interaction building blocks, patterning and shaping actions

***Establishing contact***. As shown above, caregivers were instrumental in introducing objects to very young infants who thus far are unable to approach or handle them on their own. Often caregivers took their cue from the infants' behavior: either following up on infants' gaze or action impulses, or, conversely, in trying to divert them out of their current state (e.g. pain) caregivers moved to establish contact between the infant and an object to engage with and build up a shared activity around it.

**Figure 3:**
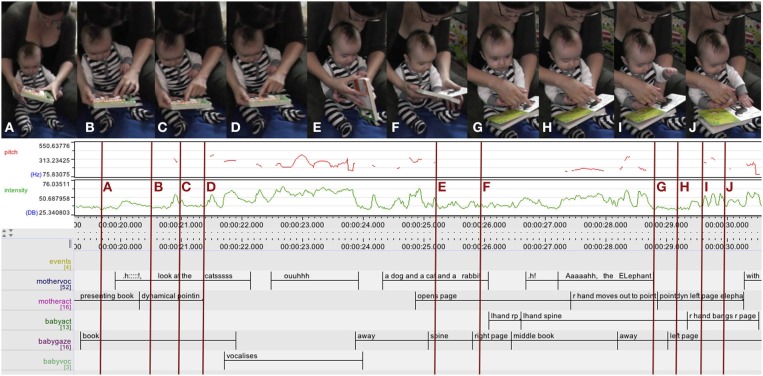
**Mother multimodally presenting a book, holding it within reach of her infant: Introducing the book to the infant (A), marking the animals on the title page by dynamical pointing and vocal labeling (B–D), opening the book with the infant attending (E,F), more dynamical pointing drawing the infant's attention (G,H), who subsequently acts on the book (I,J)**. Below the camera stills, an ELAN analysis detail documents, from top to bottom: audio traces (pitch in red and intensity in green), and annotation tiers. Tier label abbreviations used (from top to bottom): mothervoc: mother vocalizations, motheract: mother (manual) actions, babyact: infant actions, babygaze: infant gaze, and babyvoc: infant vocalizations. r: right, l: left.

In the example shown in Figure 3 the mother visually presents a book to her 4-month-old son, who is sitting between her legs leaning against her, and puts it in his reach. She starts with a sharp intake of breath indicating surprise (“.h”) (Zukow, 1982), then, pointing dynamically by moving her left index finger up and down over the pictures of the book cover, follows this up with “Look at the cats,” while the infant is looking at the book continuously. (For transcription conventions see glossary).

As shown in this example, establishing contact between infant and object often involved visual presentation, ranging from static “offering,” placing an object into the infant's view and reach, to more dynamic actions including “animating” the object, such as moving it to and fro, looming, or acting on the object. In the case of books, which were seldom animated by mothers, this prominently included performing dynamical pointing gestures, as in the example above. In addition, caregivers produced a number of different vocalizations ranging from general and unspecific exclamations of surprise (“.h”), via imperatives (“Look!”), questions (“What's that?”) to specific labels for objects or object parts (“a book!”), and content such as pictures (“an elephant!”). Among these, the most frequently used in the dataset was a sharp intake of breath indicating surprise (“.h”) combined with raised eye brows, wide eyes and open mouth.

Functionally speaking, caregivers are doing two things at once. First, they are capturing and directing the infant's attention, often utilizing the auditory domain to highlight and mark the visual presentation of an object. Second, they are making an object available to the infants to interact with “as a unit”—in this case the book itself or one of its parts. Such actions actively foreground—or even create—the object for the infant to interact with “as a unit” by “carving it out” against the background and various other ways to parse a scene, compare Zukow-Goldring's notion of “educating attention” (Zukow-Goldring, 1997, 2006, 2012).

Thus guiding the infant's attention and foregrounding or “carving out” “building blocks” to interact with, are two partly overlapping processes. They often involve performing a variety of activities composed of various strands of actions, which appeal to one or another of the infant's modalities and which can either be used (a) in close succession or (b) simultaneously, adding one on top of each other combining them into a complex multimodal action. It is especially this multimodal structure of the activity, in particular invariant relations across modalities, which provides infants with opportunities to extract coherent perception and action units (Zukow-Goldring, 1997; Bahrick and Lickliter, 2012).

***Carving out interaction building blocks and embodying meaning***. Book sharing, with its wide range of semiotically rich materials, physical spine-and-page-structure, pictures, spoken words, printed text, rhymes, narratives and referential acts is mostly about learning about, sharing, and negotiating “units” or “building blocks” to interact with, which form the public cultural interaction space. That is, these book related actions are very similar to “guiding attention and making objects available for interaction” described above; only many of the “units” forming the cultural interaction space are more abstract and are not directly graspable. Children become familiar with those “units,” how they relate to each other (pictures to pictures, words to words, pictures to words), and how all of these potentially map onto actions and relations in the world outside, and above all how to jointly manipulate and act upon them.

So how is book sharing practiced with an infant, who is preverbal, does not yet understand the referential character of pictures (DeLoache et al., 2003) and—supposedly—does not have command of joint attention either? While, as described above, the books designed for infants highlight particular physical properties adapted to their sensorimotor needs and interests, book sharing even at an early age is not at all restricted to interacting with an “interesting stimulus” or “object for manipulation.” Instead, young infants already encounter the whole range of book sharing actions.

In Figure 3 the mother is sitting on the floor supporting her 4-month-old infant boy between her outstretched legs. Throughout, she is closely following the prototypical book sharing protocol: reading out rhymed text, accompanied by additional pointing and labeling, as well as making comments relating the story to the infant's life. On his part, the infant is intently looking at the pictures, his gaze drawn through dynamical pointing, and from time to time acts on the book, either by banging or grasping the pages, which gets transformed into page turning with the support of his mother.

Neither is the infant in this interaction merely exposed to an arbitrary set of interesting stimuli and action affordances, nor does the mother blindly follow the cultural conventions. Rather, at key points in the activity, the mother is making selected parts and aspects of content and the overarching narrative accessible to the infant, making them meaningful to him through embodying and enacting them and giving them patterns of affective salience and arousal.

**Figure 4:**
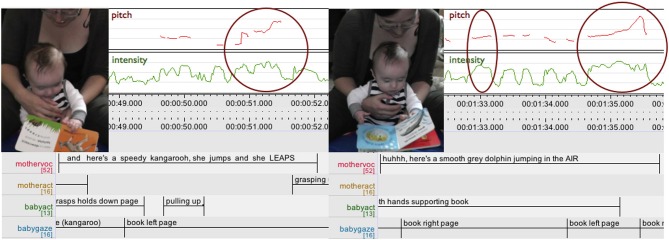
**ELAN analysis detail showing pitch (red) and intensity (green) curves**. The mother is reading a picture book about animal actions to her 4-month-old son enacting the essence of “leaping” and “jumping” (a rising motion) through a rising intonation contour (highlighted).

Figure 4 shows the mother making characteristic animal actions “come alive” and accessible to her 4 month old son through enacting the essence of “leaping” and “jumping”—a rising motion—through a rising intonation contour “This is the speedy kangaroo, she jumps and she LEAPS,” “here's a smooth gray dolphin jumping in the Air.”

Whereas in the above example the enactment takes place solely within the action medium of speech—typically utilized in picture book sharing—there are also much more extensive and thorough forms of enactment and embodiment.

**Figure 5:**
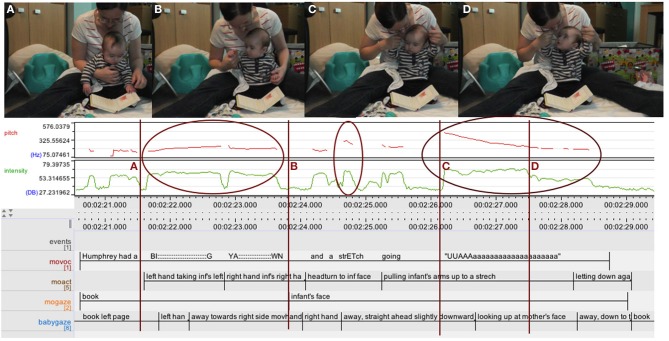
**ELAN analysis detail showing pitch (red) and intensity (green) curves**. Mother enacting and embodying a “BI::g YA:::wn and a STREtch” vocally and through acting on the 5-month-old infant's body (highlighted). Upper case letters **(A–D)** map upper row stills to ELAN time line.

In Figure 5 the mother tells her by now 5-month-old son about baby Humphrey having “a BI::g YA:::wn and a STREtch, going ‘UAAAHHH.”’ First, she utilizes prosody again, drawing out the words “BI::g YA:::wn,” thus temporally expressing the extension of “bigness” and at the same time already enacting the yawn. But then, as the text itself goes on to onomatopoetically illustrate the yawn “going UAAAHHH” she adds another layer: turning to the infant, grasping first one hand and then the other and gently pulling them into a stretch while performing the yawn, she is embodying and enacting the meaning directly with the baby's body.

In this case expressing “meaning” is no longer simply “talking about” something or “depicting” something but rather encompasses fully realizing the action itself. Only that in this special case the action of yawning and stretching, referenced in the book, is now happening in a different context than it usually would, i.e., when the infant is tired or being put to bed. Rather, this context is created and defined by the book. And as the mother is gently acting on her infant's body, taking him through the motions of stretching and at the same time performing the yawn, mother and infant closely share the meaning and the action in the sense of taking part in and realizing it together (Alač, 2005; Zukow-Goldring, 2006, 2012; Zukow-Goldring and Arbib, 2007).

***Patterning actions and shaping actions into action arcs***. Describing how objects or rather “units for interaction” are carved out to form the building blocks of a shared meaning and action space covers only one aspect of how such a space is created. This section will explore how the actions the partners perform are themselves structured in the course of interaction, highlighting the dynamic form of the jointly structured interaction space.

Two aspects of “structuring of actions” can be distinguished: The first is the temporal patterning, punctuation, and “chunking” of actions, also leading to the creation of “events” in the flow of action (Nomikou and Rohlfing, 2011). Examples include: the rhythmic multimodal performance of a monkey noise (“Ooh-Ooh-Ooh-Ooh-Ooh”), the marking and highlighting of action parts by exclamations (“.h!,” “Look!”), the labeling of action parts (“now we TURN the page”), and direct invitations (“Can you turn the page?”). Second, beyond patterning and chunking, caregivers structure actions by continually shaping parts of activities into bigger or smaller dynamic “action arcs” with a beginning, build up, climax, and resolution (compare Brazelton et al., 1974; and notions of “vitality contour” Stern, 2010; “narrative” or “shared project” Delafield-Butt and Gangopadhyay, 2013; Trevarthen and Delafield-Butt, 2013).

**Figure 6:**
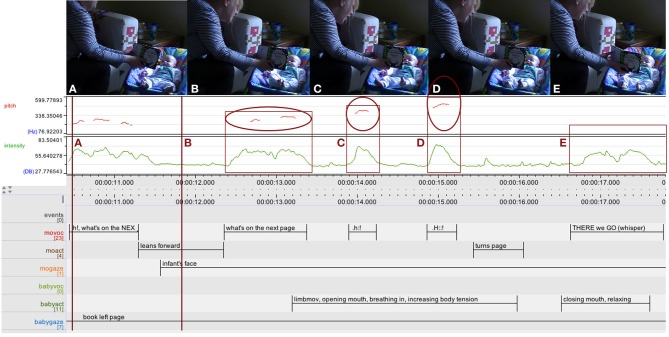
**ELAN analysis detail showing pitch (red) and intensity (green) curves**. The mother is building up an action arc through surprise exclamations of increasing intensity and pitch before releasing the tension through a quick page turn. Her 3.5-month-old infant is responding with increased movement, body tension, mouth dropping open and sharp intake of breath before relaxing again. Upper case letters **(A–E)** map upper row stills to ELAN time line.

To illustrate this we will look at the example of page turning (Figure 6). The mother sets the stage by drawing attention through the surprise exclamation “.h!” and announcing the action of page turning with the question: “What's on the next page?” Then she starts developing the action arc: leaning forward, repeating the question followed by two more “.h!” surprise exclamations of increasing intensity and pitch, she builds up tension which is mirrored in the growing arousal of the infant, indicated by her increasing movement, body tension, and facial expression, culminating in her mouth dropping open and a sharp intake of breath just before the climax. After a short hesitation—drawing forth the tension still further—a sudden quick page turn releases the tension and the arc levels off and comes to a close in a soft, whispered “There we go”, coinciding with the infant relaxing and closing her mouth again.

This shaping of action arcs is found across all kinds of actions and at different levels and multiple timescales within an activity, nested into one another. At a high level, the activity of book sharing as a whole can be considered as an “overarching” action arc structure defined by the physical arrangements of the pages to be turned from cover to cover as well as the organization of the narrative. A smaller scale action arc is defined by each double page, the unit visible at a given time, and often structured by a (rhyming) pair of lines, the first ending in a slight rise continued in one breath (enjambement) to the second one, and coming to a close in a fall in pitch and intensity. At the basic level, action arcs re-occur with any interaction unit, be it the turning of the page - itself a literal rise and fall, labeling of a picture, posing of a question, etc. Relevant words were typically placed at the peak of an action arc, and infants often looked at the caregiver's face at the peak of an action arc, as well as in a pause after an action arc's closure.

#### What about the role of the infant?

To what extent do infants actively participate in early book sharing interactions?

As briefly discussed above, it was often the infants' behavior which was prompting the caregiver to introduce an object into the interaction, which—in case the infant let him- or herself be engaged—then led to a shared object activity. Such “active interest,” that is, staying content and maintaining attention on the activity might already be considered as a form of “active participation.” Though at this age attention could easily be drawn especially by moving stimuli and also easily wandered away from time to time, infants were already able to some extent to actively control their gaze and hence their engagements. That the shared activity indeed requires an active contribution on the part of the infant became evident from cases when they withhold participation—which did not only happen when they got fussy, but also when they lost interest and kept looking away—and then there simply would not be any shared activity.

When successfully engaged, infants typically were alert and showed “serious intent” with knit brows and widely opened eyes, the type of engagement Piaget (1962) described for the adaptive mode of being absorbed in—and letting oneself be “in-formed”—in object exploration. Thus—at least for the youngest infants in the study—this shared activity looked somewhat different from other social interactions (e.g., social games) of the same infants at the same age, where more explicit expressions of joy such as laughter were observed.

However, even though not a single case of laughter in relation to a book was observed before 6 months, there was some affective communication going on in book sharing at this age: besides serious intent, a neutral expression, and occasional cases of overall fussiness, there were several instances of infants and caregivers engaging in a mutually attuned build-up of arousal in which infants showed great excitement through their bodily movements (e.g., the example of page turning discussed above, see Figure 6). Later, from around 6 months, laughter and a whole range of facial expressions were observed in an intricate emotional interplay going on between book or story, mother and infant (see Section “Ecologies in transformation”).

While caregivers significantly shape book sharing activities with 3–4 month old infants by guiding attention, inviting and scaffolding actions, infants actively participate by showing “active interest” and being responsive, amenable to their caregivers lead, letting their attention and actions be guided, and readily accepting the caregivers' invitations to engage with objects offered (De Barbaro et al., 2013).

Young infants also showed active participation in a more conventional sense in their active movements, especially manual object manipulation as far as it lay within their range of action. Whenever possible, such actions—e.g., getting hold of the edge of a page—were interpreted by the caregiver in terms of the culturally established book sharing framework (“Do you want to hold the book?,” “Can you turn the page?”), and shaped it into the frame of the book sharing activity as far as possible. These actions, however, also sometimes got in the way of the activity, especially when they could not be made to fit the book sharing frame, as when infants would not let go of a page and their own actions became their primary focus of attention (see Section “Ecologies in transformation”).

#### The interaction unfolding in the interplay between infant, caregiver, and object

After discussing the roles of mother and infant separately let us now look at one example in more detail in order to see how infant, caregiver, and artifact come together and how—out of this interplay—an interaction arises.

**Figure 7:**
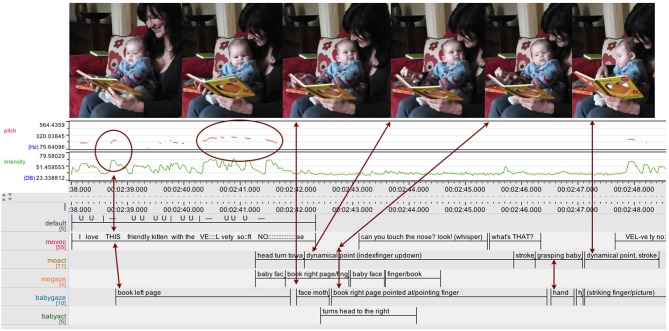
**ELAN analysis detail showing pitch (red) and intensity (green) curves**. This book sharing interaction at 4 months unfolds as smooth interplay between the actions of caregiver and infant: the infant's attention is drawn by pitch (“THIS,” arrow on the left), and after moving through an action arc looking up at mother's face, the infant's gaze is drawn back to book through dynamical pointing (arrow on the right).

In this 13 s sequence (see Figure 7) the mother is sitting on the couch with her 4-month-old boy sitting on her knee, facing away from her. Both are looking at an open picture book featuring brightly colored cat pictures and “touchy-feely” textures, which the mother is holding in front of the infant. The sequence begins with the mother rhythmically reading out a line in verse: “I love THIS friendly kitten with the VE:::Lvety so::ft NO::::::::se.” thus turning it into a two arc structure: the first arc is dominated by the deictic “THIS” which—with a sudden increase in intensity and a slight ascend in pitch—stands out as a single accentuated peak (accompanied by a slight movement of the left thumb). Thereupon the infant focuses more closely on the left page of the book. The second arc is a more pronounced, with a gradual rise in pitch peaking in “VEL-vety” followed by a slow fall in pitch and a gradual decrease in the intensity of the mother's vocalizing, during which she turns her head toward the infant. After his mother's turn toward his face, just as she arrives at the end of an elongated, soft “NO::::::::se” forming the coda of the action arc, the infant turns his head and elevates his gaze toward his mother's face. As his gaze arrives at her face with a slight delay, her gaze has already moved on to the next page, where her right index finger is now performing a dynamic pointing gesture moving up and down on the velvety textured nose, and the infant's eyes follow there soon after.

There is a sustained social interaction going on revolving around an object. Both mother and infant—acting as autonomous agents—co-regulate each other and the activity—at the same time also shaped by the object and the cultural activity frame—in ways that sustain the interaction itself (in the sense of De Jaegher and Di Paolo, 2007). The interaction is asymmetric with the infant's attention and gaze responding to and following the mother's (object related) actions and the mother guiding the interaction, checking back with the infant and adapting her actions to the infant's response. The interplay of actions has an overall smooth and orderly quality, even though the infant is slightly lagging behind in time; still the order of events in the activity is retained and meaningful for the participants, as the actions of each of them effectively serve as an affordance to the other's next action (Zukow-Goldring, 2006, 2012; Rączaszek-Leonardi et al., 2013). The infant's actions are also recognizable to the mother as turns in the context of a (culturally structured) conversation (Schegloff, 2007). The mother interprets and shapes the spontaneous behaviors of the infant to fit the cultural frame.

Like the earlier example interaction involving page turning (see Figure 6), this interaction is organized into action arcs, again clearly illustrated by the intonation curve (pitch and intensity). The relevant deictic “THIS” is placed at the peak of the arc; the infant shifts his gaze at that peak, as well as in the pause after the closure of the arc after “NO:::::::::se.” It is well known from the literature on infant directed speech that the rise in pitch—approaching the peak of the arc—makes it more likely that infants shift their gaze and is often used as an invitation for turn-taking. (Ryan, 1978; Stern et al., 1982; Ferrier, 1985; Papoušek et al., 1991) As infants and caregivers repeatedly move through action arcs together, they co-regulate and share arousal and excitement, as well as act out and experience the structure, shape, and dynamics of actions together.

### Ecologies in transformation: sketching a developmental trajectory of book sharing over the first year

Over the first year, the quality and dynamics of book sharing interactions underwent considerable change in tandem with motor development, amounting to transformations of the whole ecological setting including spatial configurations the strategies and behavior of the caregivers as well as the objects used. Some aspects of these changes have already been described in the first section, as they became manifest in gross measurements on the population level: book sharing episode durations slightly increased until 6 months, then sharply declined at 7 months. From around 6 months on, solitary interactions emerged and became the dominant type of book interactions at 8 months until social book sharing took over again at 10 months finally reaching a balance at 12 months (see Figure 1). These results closely match a series of qualitative changes observed in the course of the longitudinal study. This section will sketch a developmental trajectory of book sharing over the first year based on these changes. For this purpose, the data samples are pooled into four age groups in accordance with the newly observed interaction qualities in each period:

1. 3–4 months: early coordinated interactions with infants actively engaged but following mothers' lead cued by local dynamical events (described in previous parts).
2. 5–6 months: richer interactions with increased infant participation and more fluent attention coordination, including (a) infants shifting their gaze back to the book without being cued, and (b) interspersed affective communicative exchanges related to the book.
3. 6–9 months: social book sharing interactions turning largely into solitary book exploration with attention to own object actions, paralleling infants' new autonomous object manipulation, posture, and locomotion.
4. 9–12 months: reconstituted social book sharing: infants effectively integrate autonomous object actions—which become increasingly conventional—with the socially shared activity.

Each sub-section begins with a description of the newly observed interaction qualities in terms of the infant's activities as well as the overall ecological setting. Selected example episodes are then described and analyzed in more detail to explore and discuss attention and action coordination processes. For an overview of the changing characteristics of book sharing over the first year of life see Figure 12.

#### 5–6 months: an early peak at social book sharing interactions

**Figure 8:**
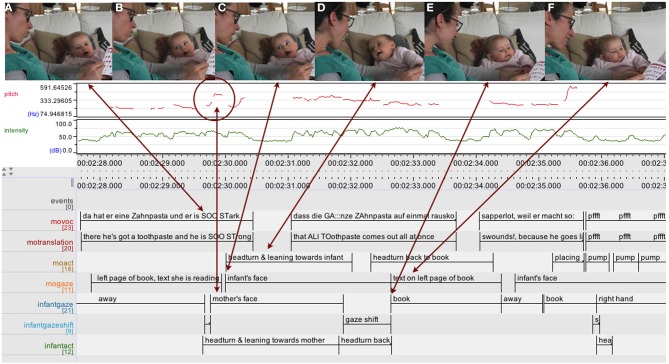
**ELAN analysis detail of book sharing interaction with 6-month-old infant sitting at a 90° angle on mother's lap**. Infant and mother looking at book together **(A)**. Infant looking up at mother's face in conjunction with salient vocal event at **(B)**. Affective communicative exchange with mutual reinforcement **(C,D)**. The infant's gaze spontaneously returns the book **(E)**, before mother's gaze returns there as well **(F)**.

**Figure 9:**
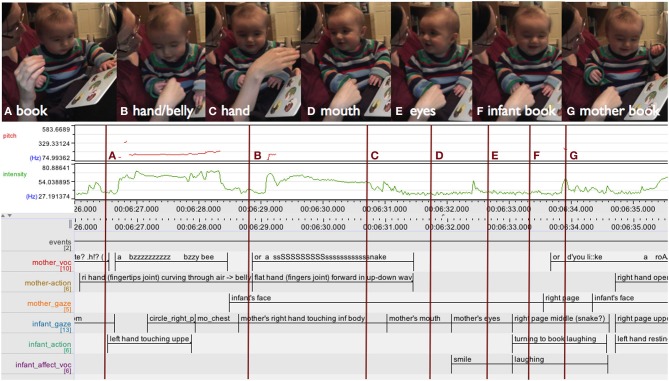
**ELAN analysis detail of book sharing interaction with 6-month-old infant sitting at a 90° angle on mother's lap**. Mother using extensive voice and hand acting to illustrate animals and animal sounds (“a bzzzzzz bzzzzy bee,” “a ssSSSSSSSSSssssssssssssnake,” **A–C**). Infant gaze alternating between book, hand, mouth, and eyes (gaze targets inscribed on still images). After communicative affective exchange **(D,E)** spontaneously looking back to the book **(F)** before mother shifts her gaze back there **(G)**.

From 5–6 months, the 2 months immediately following the early phase described in the previous sections, book sharing activities became richer, smoother, and more sophisticated in parallel with the infants' developing motor and attention skills and the increasing routine and attunement between the partners. During active participation infants used manual manipulation more extensively, showed improved aim when grasping pages, and their page flipping became more fluent. The repertoire of book interactions was extended by the addition of newly emerging actions, motor schemes such as banging, rapid opening and closing of the fingers (“scratching”) on the surface of the pages, and mouthing objects (which also began to have slightly disruptive effects on the otherwise smooth interaction). Still, these actions were largely shaped into the cultural frame by caregivers. Coordinating and switching attention between object and caregiver was performed more easily and effortlessly: infants now followed the caregiver's lead more fluently, with faster, better aimed gaze shifts from the object to the caregiver's hands or face—following his or her voice—and then looking back to the book again spontaneously, without necessarily being prompted by local, dynamical events created by the caregiver (see Figures 8, 9 below).

In accordance with infants' improving postural control and new ability to maintain a sitting position with only slight support, spatial configurations with the interaction partners facing each other at a 90° angle became more frequent. At the same time, mothers less frequently acted on the infants' body (putting them through the motions of a specific action); rather, mothers used their own body and voice, especially their hands, to enact meaning and perform lively visual demonstrations (including the beginning use of baby signs). In line with the increasing frequency and skill of infants' object manipulations, books with touchy-feely textures and attached graspable objects became prominent, as did books made of real paper with audio-haptic crinkle.

Figures 8, 9 illustrate the new quality and range of book sharing interactions at 5, and especially 6 months with a focus on co-ordination of attention and of action.

In the first example (see Figure 8) the mother is sitting on the couch cross-legged with her 6-month-old daughter placed at a 90° angle in the hollow formed by the mother's left leg with her back supported by the mother's left thigh and a sofa cushion. They are both facing a small square paperback “Mr. Men and Miss Little” book with thin paper pages which the mother is holding. Immediately after a sharp rise in the intonation curve (“er ist SO::stark” [“he is SO::strong”]), the infant turns her gaze upwards toward her mother's face, who in turn responds with an eye-greeting and a more pronounced facial expression and affective intonation. They share and reinforce each other's expression of surprise and amazement in voice and facial expression before first the infant and then the mother turn their gaze back to the book again.

In the second example (see Figure 9), the mother and her 6-month-old son sitting on her lap at a 90° angle are sharing a book about animal noises and have just arrived at the last page. After setting the scene by “Who's your favorite?” the mother starts curving her right hand with the fingertips pressed together through the air toward the infant—accompanied by “a bzzzzzz bzzzzy bee”—with her eyes fixated on the infant, who is still involved with the book, his left hand reaching for and touching the animal picture on the upper right corner of the right page. When the mother's hand finally touches the infant's belly, he turns his gaze and head to her hand and begins tracking her hand as she starts moving it with her fingers joined side by side in up and down waves acting out “… or a ssSSSSSSSSSssssssssssssnake.” As the mother concludes her enactment of the snake, the infant looks up first at the mother's mouth and then at her eyes, beginning to smile. He then turns his gaze to the book again, his smile broadening, shortly after being followed by the mother returning her gaze to the book.

***Infants' attention coordination becoming more fluent and guided by routine***. In both examples the infant is responding to an aspect of the mother's behavior related to the book, e.g., the intonation curve going up as part of the mother's interpretation of the narrative. In a previous example at 4 months (Figure 7), the infant was responding to and following the mother's salient actions but kept lagging slightly behind and so the mother's gaze had already moved back to the book by the time the infant had shifted his gaze to his mother's face. In contrast, this time the eyes of mother and infant meet, facilitated by the 90° configuration and the infant's more fluent movement. The infant thus elicits a communicative exchange of affect, including mutual acknowledgement and reinforcement. Also in contrast to the previous interactions, in both these cases it is now the infant who first turns his/her gaze back to the book again, before the mother does….

While infants, despite their growing motor skills, are still unable to autonomously move in and explore the world of objects, they are now turning their gaze and head more fluently from book to the caregiver's hand or face and back again. They do so spontaneously, without necessarily being cued by dynamical movements, but arguably guided by routine, at times even arriving back at the book first, taking the lead in coordinating attention. Thus, within these interactions, infants demonstrate a basic understanding of the activity as shared and of the spatio-temporal structure and format of the book sharing activity at hand. The examples at 6 months also invite us to consider how small changes in the temporal dynamics of the interaction can lead to profound qualitative shifts as infants' more fluent gaze coordination enables episodes of affective communicative emotional exchanges, and thus increase the infants' ability to effectively shape the interaction dynamics of the book sharing activity.

***Interspersed affective communicative exchanges related to the book***. Whereas at 3–4 months, infants showed “serious intent” when engaging in book sharing interactions, along with these novel communicative exchanges, infants now show pronounced affective exchanges.

**Figure 10:**
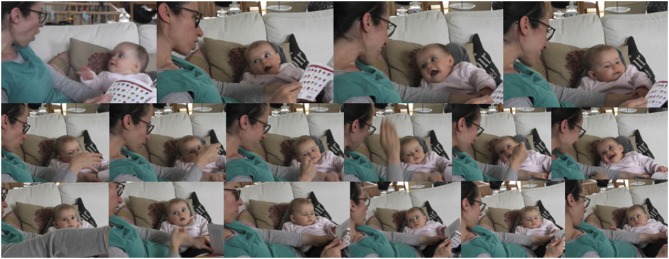
**Book sharing interaction with 6-month-old infant sitting at a 90° angle on mother's lap including extensive voice and hand acting**. Still images showing sequence of emotional exchanges: going in rapid succession and hand in hand with the mother's tone of voice and movement when narrating the story, the infant moves from surprise, amazement, to amusement, and from being staggered to concern and sadness.

While the mother narrates the story, in the short span of 5 min the infant displays and moves through a whole range of emotions in rapid succession, in concordance with the mother's tone of voice, her gestures and movements: from surprise and amazement to amusement, and from being “staggered” to concern and sadness (see Figure 10). The emotions build up and develop in the flow of the interaction. In response to the mother's voice and actions the infant looks up to her face with an expression of surprise, for example after an abrupt rise in pitch contour in “SO::strong,” the mother takes up her daughter's expression and responds to it with widely opened eyes, raised eye-brows, and a sharp intake of breath indicating surprise (.h). She then repeats the passage that drew her daughter's attention to her “SO::strong,” again with exaggerated pitch contour, reinforcing and further shaping her daughter's emotion, thus acknowledging and reinforcing each other (compare Stern, 1985; Jensen, 2014 this issue).

So they were moving through the emotions together without however seeming to be seriously upset or sad. Importantly, these communicative exchanges are situated in the book sharing context, immediately following and leading back into attentional engagement with the book. Thus, the exchanging of emotions appears clearly linked to the book, and even to constitute a jointly relating to and negotiating “about” the book (see general discussion below).

#### 6–9 months: shifting attention to object exploration

**Figure 11:**
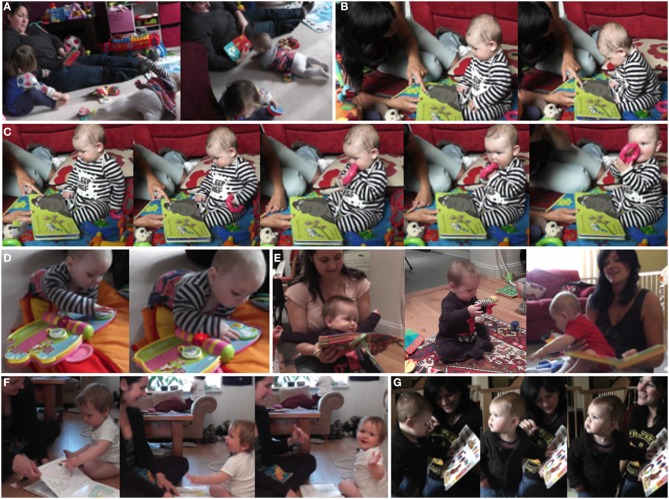
**(A)** 7-month-old infant initiating book sharing by crawling toward the book. **(B)** 6-month-old, sitting freely, focusing on mother's dynamical pointing and further closing in. **(C)** the 6-month-old in the same interaction getting distracted after accidentally touching and subsequently grasping and mouthing a toy ring. **(D)** 7-month-old absorbed in solitary play: correctly operating interaction device resulting in music and blinking. **(E)** 9-month-olds escaping from the book sharing activity despite their mother's attempts to engage them. **(F)** 11-month-old proactively performing appropriate actions for “Pat the bunny”: putting his finger through the ring, sharing affect with his mother while making dolly's ball squeak by banging on it, and “waving bye-bye” directed at the researcher, thus connecting the book sharing context with the visitor context. **(G)** Mother naming, pointing at, and signing “bird,” infant turning head looking out of the window while mother is still involved with the book, before mother turns her head recounting how they saw a bird out there the day before.

During the next few months, however, roughly in the period between 6 and 9 months of age, the interaction dynamics of infant-caregiver-object interactions underwent a significant transformation and the course of the developmental trajectory took a sharp turn: infant-object-caregiver interactions decreased in number relative to solitary book exploration, and book sharing interactions showed a considerable decrease in duration and appeared generally less smooth compared to the period before, in spite of the infants further developing their capacity to sustain attention (see Figures 11B,E).

These changes occurred in a period when the infants' developing strength and postural control allowed them to adopt and maintain a stable sitting position for longer periods of time, enabling them to reach and grasp and bimanually manipulate objects without falling over. Also, many infants at this age started locomoting by rolling and (“army”) crawling, and actively initiated interactions in a clearly visible way. The 7-month-old girl in Figure 11A for example, noticing a book sharing interaction taking place between her mother and sister, glances over her shoulder, rolls over from back to belly, and crawls across the room toward the book (still held by her mother but abandoned by now by her older sibling), thereby prompting her mother—albeit without explicit social signals—to start a book sharing interaction. Infants were also better able to focus and maintain their attention—see the 6-month-old boy in Figure 11B intently watching his mother's stroking a texture and closing in to see better. However, they were also more likely to quickly terminate interactions as their newly developed autonomous object exploration and locomotion activities drew them into new attentional engagements. In Figure 11C the same 6-month-old, after sitting back up again, accidentally touches a toy ring, subsequently grasps it and—with his eyes still on the book—brings it to his mouth, at which point his gaze is finally distracted away from the book and he becomes pre-occupied with exploring the ring, bringing the book sharing activity to a halt.

In this period, facilitated by the now stable sitting posture, infants got at times deeply involved with objects, e.g., banging, mouthing and manipulating books or other objects in solitary play to the extent of seemingly ignoring people: having escaped from a book sharing interaction after barely 2 min the boy in Figure 11E engages in manipulating a single object for nearly 6 min without interruption immediately afterwards. Infants did, however, from time to time look up at people's faces, e.g., when introduced to an object, or in what might be early forms of instrumental looking: after having pushed a book out of reach, a 6-month-old girl lying on her belly turned her head up to her mother's face and vocalized.

These changes were also reflected in the caregiver's behavior: they were now often content to leave the infants to their solitary play. When they did try to engage them in book sharing, their efforts of directing attention became more vigorous: for example, they called their infant's name repeatedly with increasing intensity to get the infant's attention and resorted to acting on the infant's body again, but now in an exaggerated fashion to keep the infant entertained. Caregivers also adapted by changing the situational context: for example, they tried to engage infants in book sharing interactions before bedtime, when infants are already tired, or changed the spatial configuration by placing infants on their lap, thereby actively constraining their action possibilities.

Books chosen by caregivers during this period had more interactive elements: in addition to the touchy-feely textures, flaps, and small graspable objects, they now included buttons producing various animal noises and moveable parts set on massive plastic pages eliciting blinking lights and nursery rhymes when operated correctly (Figure 11D). Thus, books are designed to invite manual exploration and multimodal interaction, drawing in infants now able to approach and engage with books on their own. On their part, caregivers included these highly salient object interaction opportunities in their social interactions to make them more interesting again to their infants with mixed results (Figure 11E).

#### 9–12 months: putting books, caregivers and world back together

At 9–12 months, infants continued to engage in many solitary book interactions, but in contrast to the previous months, when they had primarily been exercising various motor schemes, banging, scratching, mouthing the book, as well as bimanually exploring books, they now started showing many more behaviors associated with conventional book interactions such as sitting still and looking at the pictures, turning pages, opening flaps, pointing at pictures, touching textures, and vocalizing.

Also in contrast to the previous period, the proportion of social book sharing episodes in relation to solitary ones increased again. Both solitary and social book interactions showed considerable variations in duration. Although the majority of the interactions were short, at times infants engaged in book interactions for extended periods lasting up to 7 min, as well as chained several episodes together into much longer lasting book activities. For example, they would ask for another round of looking at a specific book several times in a row, or, according to the mothers' reports, entertain themselves during car journeys by looking at books and turning pages for extended periods of time.

Book sharing episodes, even short ones, encompassed an increased number of action turns and showed a new quality and a larger degree of integration between interactions with the caregiver and with objects, between book and world and across time and space. Infants now more actively integrated manual object actions into their social engagements (e.g., approaching the mother with a book, laughing) and, when engaged with objects, now integrated social interactions (pointers, requests…), which may or may not include gaze alternations. Moreover, they were now actively bidding for and directing others' attention.

Infants now moved pro-actively in the spatiotemporal attention-action framework of an activity: spontaneously performing appropriate actions in a specific context independent of temporal order, e.g. performing an action corresponding to a specific book page (“pat the bunny,” “put the finger through mommy's ring,” “wave goodbye”—see Figure 11F), and were also able to anticipate what came next. The infants' actions extended much further over space and time, between the book and the world, while still being part of and coming back to the shared activity. For example, a boy interrupted his immediate engagement with the book, ran off and found the object depicted in the picture book and returned to mother and book. Or when the mother in Figure 11G is pointing out and signing “bird” referring to the picture in the book the infant is turning and looking out of the window. Not realizing this, the mother first finishes her signing, and then herself turns to look to the window recounting how they had encountered a bird there on the previous day.

### Conclusions, general discussion, and outlook

Our 3 main findings were:

1. Infant-caregiver-object interactions occurred from as early as 3 months. They unfolded as joint, mutually coordinated activities depending on the active contribution of all participants, and involved different kinds and degrees of attention as well as action co-ordination between co-participants and object.
2. Over the course of the first year the quality and dynamics of book sharing interactions underwent considerable change in tandem with motor development, amounting to transformations of the whole ecological setting: book sharing episodes became more fluent and sophisticated until 6 months, after which there was a marked decrease in duration whereas solitary interactions became dominant, as infants developed novel postural, manipulation and locomotion skills and their attention shifted to learning to effectively act on the object world. Subsequently, social book sharing interactions resurfaced in the period from 9 to 12 month, showing novel qualities, as infants began to effectively integrate manual object actions—which also became increasingly conventional—within the socially shared activity.
3. Our understanding of the emergence and development of triadic interactions and co-ordination and sharing of attention and action can be enhanced by looking at the larger ecological context, especially at the hitherto overlooked early period from 3 to 6 months and how shared spaces of meaning and action are structured together in and through interaction, creating the foundation for cooperation and cultural learning.

#### Development of triadic interactions

With regard to various theoretical accounts concerning the development of triadic interactions our observations suggest that:

Interactions with objects and interactions with people are not separated during the first year as often suggested in the literature (Bakeman and Adamson, 1984; Tomasello et al., 2005). On the contrary, at around 3 months when infants' interests start to reach beyond the dyad but they lack the means to effectively interact with the material world on their own yet, objects are introduced by their caregivers in the context of social interactions.

Instead of a late, sudden appearance of triadic interactions at the end of the first year, we report a much more gradual development (compare Striano and Reid, 2009; De Barbaro et al., 2013)—albeit following a non-linear trajectory, characterized by an apparent dip after around 6 months followed by a recovery starting from 9 months; this would also explain why the earlier interactions have been largely overlooked in the literature.

The qualitative changes in the period between 9 and 12 months need a more differentiated conceptual framework as many of the criteria for triadicity—active contribution of the infant, co-ordination of attention and action between caregiver and object, etc.—already seem to be met by earlier interactions. Key notions need to be clarified and re-conceptualized, including: the nature of the infant's active contribution, infants' coordination of attention/orientation actions in relation to their coordination of manual actions and in particular the concept of joint attention.

**Figure 12:**
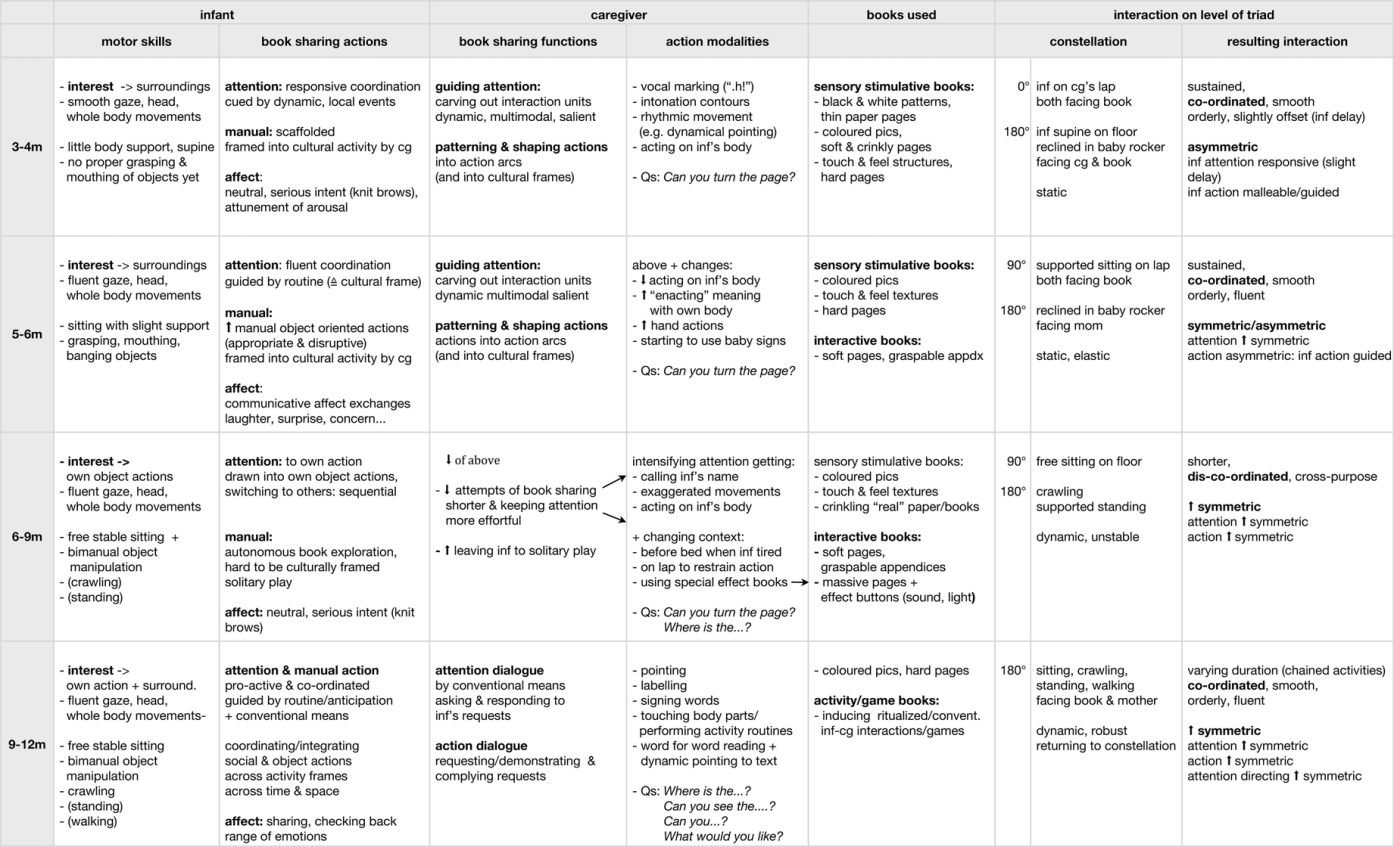
**Ecologies in transformation**. The table gives an overview of book sharing as it changes over the first year. The columns list relevant characteristics for the respective participants: infant (inf): motor skills and book sharing actions sorted in attentional, manual and affective; caregiver (cg): book sharing actions in terms of function and modalities they are implemented in; books: type of book used; and for the interaction as a whole: the spatial configuration of the participants and the quality of - the resulting interaction. The rows list the pooled age groups (3–4, 5–6, 6–9, 9–12 months).

***3–4 months***. At 3–4 months the infants showed active interest in the activity. They were responsive, amenable to and following the caregiver's lead, effectively co-ordinating their engagement between caregiver and object, their attention being drawn by local dynamical cues created by the caregiver (though following with slight delay) and their (rudimentary) manual actions were shaped into cultural frames by the caregiver. Thus the interaction was co-ordinated but asymmetric, smooth and orderly but slightly off-set (see Figure 12).

Accounts of infants' (lack of) triadic behavior at this early age do not begin to capture these intricacies revealed through the qualitative micro-analysis. For example, in Adamson and Bakeman's (1984) notion of passive joint engagement, the caregiver establishes and sustains the (passive) triadic interaction essentially all by herself. By turning to whatever the infant is engaged with or directing the infant's attention to a specific target, she ensures that infant and caregiver are “actively involved in the same object, but the baby evidences little awareness of the other's involvement or even presence.” (p. 1281) In early book sharing, however, the infants were clearly not oblivious to the caregivers' presence, as evidenced by e.g., their regular gaze shifts between caregiver and object, drawn by the caregiver's voice and movements. Rather, early book sharing already comes close to their description of *coordinated joint engagement* characterized by the infant being “actively involved with and coordinating his or her attention to both another person and the object that person is involved with.”

While it is arguable whether the responsive nature of the 3–4 month infant's engagement completely matches this set of criteria introduced to describe the behavior of infants 9 months and older, by 5–6 months, infants' active involvement was pronounced, especially with respect to their attention coordination.

***5–6 months***. At 5–6 months infants now coordinated their engagement between caregiver and object more fluently, and shifted their gaze back to the book by themselves without the need for a prompt arguably guided by routine. Their gaze often arrived back at the book first, thus at times leading the interaction. As faster gaze shifts led to meeting the caregiver's eyes, infants now entered into affective exchanges and sequentially coordinated these exchanges with periods of shared object involvement. Despite their improved motor skills, infants were still unable to move in and explore the world of objects on their own. In book sharing, their range of manual contributions has expanded, including both helpful and disruptive actions, which were still mostly shaped into the cultural frame by their caregivers. Thus the interaction is co-ordinated and more symmetric with regard to attention, but asymmetric in terms of action, and overall orderly and fluent (see Figure 12).

Due to the interspersed affective exchanges, the interaction already resembles Hubley and Trevarthen's concept of *secondary intersubjectivity*, characterized by integrating “acts of joint praxis” around objects with “interpersonal communicative acts” (Hubley and Trevarthen, 1979). On the other hand, infants may not show enough manual object actions yet, and alternating back and forth between shared book involvement and communicative affective exchanges sequentially (see Figure 9) may not be “integrated” enough to match the criteria again set to describe the behavior of infants around 9 month and above.

Whatever the verdict on its “triadic” status, this alternation between engagements may constitute a basic form of “joint aboutness”—jointly communicating about something—which plays an important role in secondary intersubjectivity. It is also reminiscent of a crucial notion in Liebal and Carpenter's account of joint attention: one of its central features, “knowledge of knowing together,” is held to be established via what they call “sharing looks.” These looks close the triangle of the triad, turning “not-yet-shared attention into truly joint, shared attention,” confirming that attention is shared, with the goal of bringing about “an alignment of attitudes” (Carpenter and Liebal, 2011; compare Hobson, 2005). Their account again refers to infants at around 9 months and older and was not intended to capture the behavior of younger infants. Notably, social book sharing interactions at 6 months seem to already constitute a basic comment structure, in Bruner's terms (1975), in that infant and caregiver exchange affect in relation to, or even “jointly negotiate about” the book. Thus the affective exchanges in conjunction with the joint involvement with the book, its pictures, and vocal narrative might constitute a basic form of “content” and the succession of emotional exchanges may build up toward a basic form of “emotional narrative.”

***6–9 months***. At 6–9 months, infants were actively seeking out and autonomously manipulating books, mostly engaging in solitary book exploration, with their attention primarily drawn to their own manual object actions, only at times looking up at their caregivers. Thus the social book sharing episodes were shorter, as the infants failed to keep up their engagement with the caregiver long enough to sustain the interaction. Though the interactions were now more symmetric, due to the infants' more autonomous object manipulation, they were also less coordinated, at times dis-coordinated: when their caregivers attempted to guide them, infants were frequently already involved in an action, putting them at cross purposes (compare De Barbaro et al., 2013), and their manual actions could no longer easily be shaped into the cultural frame of book sharing (see Figure 12).

Looking at the period between 6 and 9 months revealed that the configuration commonly described in the literature for most of the first year does indeed occur: there was little joint or shared action as infants were drawn into deep object involvement to the point of seemingly “ignoring people” (e.g., Tomasello, 1999). However, when looked at more closely in the bigger ecological context, the apparent dip in triadic interactions at this point is not the beginning of the story but rather is only temporary, following a period of already well coordinated infant-caregiver-object interactions.

Rather than reflecting an enduring lack of cognitive capacities, the relative paucity of triadic interactions compared to solitary book sharing interactions between 6 and 9 months can hence be understood as a change of interaction dynamics due to new achievements (developing object manipulation, posture and mobility) and accordingly shifting interests. This shift of interest toward objects has long been known in the literature (Trevarthen and Hubley, 1978; Bakeman and Adamson, 1984). To characterize it (beyond noting basic correlations with infant postural and motor development) further investigations are required at the micro-developmental level (see De Barbaro et al., 2013). The primary focus in the literature on the development of triadic interactions in terms of underlying cognitive capacities “coming on line” only later on explains why the diminished and dis-coordinated social object interactions at this age range are ignored and why the significance of early triadic interactions has been so often neglected and even overlooked (Tomasello et al., 2005; compare Reid and Striano, 2007).

***9–12 months***. At 9–12 months infants' attention and action were guided not only through dynamical cues and routines but also by indirect and conventional means (words, instructions, demonstrations). Infants' fluent coordination at this age incorporated manual object actions into social actions and social actions into manual object actions across different cultural activity frameworks, across time and space. Infants increasingly shaped and adapted their now versatile locomotion and object manipulation actions according to the conventional frame and to communicative exchanges, and were themselves actively directing others' attention and action. The episodes were of varying duration, with a high frequency of action turns, and often chained together. The interactions were mostly coordinated and symmetric, orderly and fluent (see Figure 12).

This period clearly encompasses significant qualitative changes in the interactions. Rather than appearing suddenly supposedly mediated by a newly emerging capacity of joint attention, these changes can be seen as part of a gradual development (compare De Barbaro et al., 2013), coming out of the interplay of multiple strands of development in interaction with the social and cultural environment and the entire ecology of the activity.

In order to further explore and better understand the interplay of these multiples strands of development we need to reframe, refine, and expand key notions such as (visual) joint attention to create conceptual frameworks which likewise allow for an interplay of multiple concepts capturing different aspects of the interactions, cultural activities, and their ecologies. For example, whereas the concept of joint attention, which developed in the context of experiments on gaze following and gaze checking (Scaife and Bruner, 1975), is primarily focused on the visual domain, processes such as sharing of experience, attention coordination, mutual orienting can rely on multiple modalities bound together in structured actions. The role of gaze within this interplay of modalities is only beginning to be explored in more detail (e.g., social gaze to eye-hand-coordination in caregiver-infant-object interactions Yu and Smith, 2013).

#### Jointly structuring shared spaces of meaning and action

The richness of early infant-caregiver-object interactions in naturalistic contexts invites an expansion of focus from the supposedly late emerging triadic interactions primarily associated with visual (joint) attention to studying how shared spaces of meaning and action are multi-modally structured together from early on.

The infants' situation at 3–6 months (showing interest in their surroundings but not yet being able to explore the object world on their own) makes this age window particularly interesting for learning socially (including learning “about objects and the world”), as the infants readily engage in the highly structured and experientially rich joint activities offered by their caregivers.

Book sharing is such an activity. It serves as a “container” holding infant, caregiver, and world together in a small confined space opening up possibilities for shared experience and action and fostering learning (Wood et al., 1976; Vygotsky, 1978). In pointing actions, for example, rather than having to follow a pointing finger to a distant target, the close encounters of early book sharing allow the finger pointing and the object pointed at to meet in immediate vicinity and within the infant's reach, often accompanied by salient, dynamical gestures and actual, audible contact events. The container offers a rich reservoir of—and substrate for creating—interaction structures which are easily accessible to learn from and act upon together (Shotter, 1983; Goodwin, 2013). Part of this (spatial as well as temporal) structuring is provided by the cultural book sharing framework created around and manifested in the artifact book. Not only does the book invite the infants to physically engage with it (scaffolding their manual actions), it also embodies and reliably reproduces a stable, recognizable and predictable sequence of actions. What makes the activity come alive is the caregivers' active moment-to-moment structuring as they dynamically enact and carve out “building blocks” of interaction, pattern actions, and shape actions into action arcs in dialog with the infants.

The wealth of information available in infants' natural environments has been emphasized by computational approaches in order to explain the impressive early achievements of infant learners, focusing primarily on the problem of word-reference learning (Smith et al., 2014). Also the statistical validity of social cues (caregivers' action and gaze directions) for finding and disambiguating meaning in the complex cluttered streams of objects, actions, events—and words—has been shown using statistical learning models (Frank et al., 2012). Caregivers in real world activities actively select and structure their infant-directed speech, performing “auditory packaging” closely coupled to the relevant actions, creating crossmodal invariances, thus simplifying learning by highlighting relevant aspects within the interaction (Nomikou and Rohlfing, 2011; Bahrick and Lickliter, 2012; see also Leavens et al., 2014, this issue).

The present study invites us to take a step beyond the structuring of “perceptual input,” and consider the infant's active, embodied participation and engagement in joint practices. Infants experience the activity first hand, actively seeking out and probing their environment through active vision and active touch. They are fully immersed and emotionally invested in coordinated interactions with their caregivers and the book, actively structuring shared spaces of meaning and action together. To describe this structuring in more detail we used the notion of “action arcs.” The basic arc structure with a beginning, build up, climax, and resolution is ubiquitous in physiological processes, e.g., breathing, and is fundamental to action, with different actions following different dynamic trajectories (Stern, 2010; Trevarthen and Delafield-Butt, 2013).

As infants and caregivers repeatedly move through action arcs together, they co-regulate and share arousal and excitement, as well as act out and experience the structure, shape, and dynamics of actions together. These types of co-regulation could be regarded as merely coordination of behavior with sharing of affect (Tomasello et al., 2005). However, in moving through these arcs together, sharing of affect goes hand in hand with, and is inseparable from, learning about the structure of the action: infants become familiar with the dynamic trajectories as they are led through the motions, providing an opportunity to learn about structure and dynamics of actions, about themselves, their partner, the object involved, and their relation. Moreover, they get to experience and learn about the effects their own actions have on the partner and the unfolding of the activity.

Through such immersion in participation, infants are able to learn specific routines and practices, and more generally, “ways of interacting,” following the implicit norms of their culture (Mauss, 1973; Rietveld, 2008). It also provides the opportunity to learn about other people as social agents, whose actions significantly shape the unfolding of the activity. Through being drawn repeatedly by cues and movements to the relevant locations—hands, faces, objects—“where the action takes place”—infants become accustomed to and learn to anticipate the specific sequences of action trajectories (e.g., Hunnius and Bekkering, 2010), and the interplay of gaze, hand actions, and object use—in short how people act.

Crucially, infants are learning how to learn: when to look, where to get important information, and when to join in with an appropriate action (e.g., after a rising action at the peak of an action arc). Once established as interpersonal routines, action structures lend themselves to be played with, e.g., introducing temporal variations that violate expectations (as in teasing), thus highlighting and making explicit mutual coupling and co-regulation, potentially helping to develop action coordination skills and cooperation (Reddy, 2008; Reddy et al., 2013). As active participants even in early interactions, infants become familiar with how to jointly structure activities and begin to learn how to negotiate and modify this shared structuring of activities. This skill, developed further, may be characteristic of how infants coordinate triadic interactions at 9–12 months, and crucial for cultural learning and culture creation.

### Conflict of interest statement

The authors declare that the research was conducted in the absence of any commercial or financial relationships that could be construed as a potential conflict of interest.

## Footnotes

^1^Notion by Jakob von Uexküll (Uexküll, 1921; Uexküll et al., 1956). An interpretation in contemporary terms: *Umwelt* refers to those aspects of the environment an organism can interact with—i.e., effectively perceive, distinguish and act on (= the sum of prospective functional action-perception cycles)—and which hence constitute the organism's meaningful world. This world is subjective, different organisms/subjects who have different histories and possibilities of interaction live in/enact different worlds.

## Glossary

In the micro-analytic descriptions and ELAN illustrations some transcription conventions from conversation analysis were used where appropriate. (See Zukow, 1982; Jefferson, 2004)

? Question mark: rising intonation

. Full stop/period: falling intonation

, Comma: continuing intonation

! Exclamation mark: animated tone

AIr Upper case: increased loudness relative to surrounding sound

.h Period preceding h: audible inhalation, in particular: sharp intake of breath indicating surprise

BI::g Colons: lengthening of preceding sound, the more colons, the longer.
